# The association between state direct access laws and earlier use of physical therapy among Medicare patients with rotator cuff tears: A retrospective cohort study

**DOI:** 10.1097/MD.0000000000048584

**Published:** 2026-05-08

**Authors:** Dakshu Jindal, John M. Brooks, Nicole L. Hair, Adam D. Lutz, Brian K. Chen

**Affiliations:** a Department of Health Services Policy and Management, Arnold School of Public Health, University of South Carolina, Columbia, SC; b Department of Public Health Sciences, Henry Ford Health, Detroit, MI; c Department of Health Services Policy and Management, Center for Effectiveness Research in Orthopedics, Arnold School of Public Health, University of South Carolina, Columbia, SC; d ATI Physical Therapy, Institute for Musculoskeletal Advancement, Greenville, SC.

**Keywords:** atraumatic rotator cuff tear, direct access laws, Medicare, physical therapy

## Abstract

Current literature supports physical therapy (PT) as an initial treatment for patients with musculoskeletal pain without severe trauma, but state laws can limit timely access depending on the level of direct access permitted. We assessed whether more permissive direct access laws are associated with earlier PT use among Medicare patients with an atraumatic rotator cuff tear (ARCT). We obtained claims files from 2016 to 2018 for all US Medicare fee-for-service beneficiaries with an ARCT diagnosis in 2017, using 2016 data for baseline confounders and 2018 data to capture outcomes. Earlier PT was defined in 2 ways: PT first – seeing only a physical therapist on the first shoulder-related outpatient visit within 90 days of ARCT diagnosis and early PT – seeing a physical therapist within 30 days of this first visit. Multivariable logistic regressions assessed whether more permissive PT access laws were associated with greater odds of earlier PT use, adjusting for confounders. Compared with patients in limited access states, those in unrestricted and provisional access states had higher odds of PT first (odds ratio [OR] = 1.8, 95% confidence interval [CI]: 1.5–2.1 and OR = 1.6, 95% CI: 1.3–2.0, respectively). Relative to patients in states with limited access, patients in states with provisional access had (OR = 1.2, 95% CI: 1.1–1.4) higher odds of early PT. No significant differences were observed between unrestricted and provisional access states. State laws permitting more direct access to PT may encourage earlier use of PT among patients with ARCT. Further research should investigate whether these law-associated treatment differences also improve health outcomes and lower costs.

## 1. Introduction

Musculoskeletal conditions (MSK) affect bones, joints, muscles and connective tissues, often leading to pain, disability, and high cost of care. In the United States (US), MSK conditions are the leading driver of healthcare expenditures, costing $420 billion in annual direct medical expenditures, more than all other major chronic conditions such as diabetes, cardiovascular diseases, cancer and chronic respiratory diseases.^[1,2]^ A 2024 report estimated that nearly 1 in 2 Americans experience MSK pain, which can adversely affect virtually all aspects of everyday living and work.^[3]^ Physical therapy (PT) is often considered the best first-line treatment for many patients with new MSK conditions without severe trauma.^[4-7]^ As a conservative treatment, PT can reduce pain and improve balance, functional outcomes and quality of life without the risks associated with surgery.^[8-12]^ Unlike pain medications that offer only temporary relief, PT provides a long-term approach to address the underlying causes of chronic pain.^[11]^ By preventing or delaying costly surgeries and opioids use,^[13-17]^ earlier use of PT can potentially address both rising healthcare costs and the opioid epidemic, two of the most pressing public health challenges today.

Despite these potential benefits, state scope of practice laws had traditionally prevented patients from accessing PT directly.^[18]^ Under these laws, only physicians could diagnose or treat a patient’s physical and functional limitations. Access to PT required a physician referral, potentially discouraging earlier PT use. Over the years, however, the American Physical Therapy Association (APTA), along with many state affiliates, have successfully advocated for state law changes to permit direct access to PT.^[18]^ These direct access laws varied across states, and in prior years ranged from limited access to provisional and unrestricted access.^[19]^

In 2024, Alabama became the last state to remove its limited direct access law, demonstrating continued policy interest in expanding access to PT. All 50 states and District of Columbia now have either unrestricted or provisional direct access, the latter of which imposes some limitations such as capping the number of PT visits before a physician referral is required. Despite the growing permissiveness of state direct access laws, however, there is still little empirical evidence showing direct access *law*s increased earlier use of PT to treat MSK conditions. While many studies have examined direct access to PT, only 1 study to date has specifically evaluated the impact of state direct access laws. A study by Kazis et al showed that commercially insured patients with lower back pain in states with less restrictive direct access laws were more likely to use PT first.^[20]^ However, the study findings may not isolate the effects of the laws because commercial plans often have plan-level restrictions on direct access to PT.^[21,22]^

To overcome this knowledge gap, we studied this question in Medicare fee-for-service (FFS) patients with atraumatic rotator cuff tear (ARCT), a prevalent MSK condition affecting 20% of older adults aged 50 and over in the United States.^[11]^ Unlike commercial insurance plans, Medicare FFS is a national health insurance program that offers advantages for our identification strategy. Medicare was established by the Social Security Amendments of 1965 and primarily covers adults aged 65 years and older, as well as certain individuals under 65 with qualifying long-term disability or specific serious conditions. Because Medicare FFS benefit design for outpatient PT is largely standardized nationally, using Medicare FFS helps minimize heterogeneity related to insurer-specific coverage and cost-sharing that can complicate comparisons across states. Our analytic dataset was derived from national Center for Medicare and Medicaid Services Medicare FFS administrative claims and enrollment files. While the database is national, beneficiary state of residence is recorded and was used to link each beneficiary to the applicable state direct access policy environment. We used Medicare fee-for-service claims from 2016 to 2018 to examine associations between state direct access laws and earlier PT use.

The study had 2 aims. First, when states still differed across limited, provisional, and unrestricted access in 2017, we compared provisional and unrestricted laws with limited access. Second, we then compared unrestricted with provisional access to isolate differences within the contemporary policy environment, where all states permit at least provisional direct access. Findings from this study may help inform policymakers whether more permissive state direct access laws can lead to earlier PT use, which may in turn result in better pain management at lower cost and fewer adverse effects from opioids and surgery.

## 2. Materials and methods

### 2.1. Data and sample construction

We obtained all claims data for Medicare FFS enrollees with a shoulder-related condition from 2016 to 2018. Although 2017 served as the primary index year for analysis, data from 2016 and 2018 were included to allow measurement of baseline covariates and outcomes. From these files, we identified patients with a confirmed diagnosis of ARCT. However, ARCT is not always identified during a patient’s initial visit to a provider.^[23]^ To establish the index date for this diagnosis, we identified the date of the patient’s first shoulder-related visit in 2017 and designated it as the “index date” if the patient subsequently received an ARCT diagnosis within 90 days (see Fig. A, Supplemental Digital Content). To restrict our sample to incident cases of ARCT, we excluded patients with a shoulder-related diagnosis in the 365 days prior to the index date. The diagnosis of ARCT was determined using national Classification of Diseases, Tenth Revision (ICD-10) diagnosis codes M75.100, M75.101, M75.102, M75.110, M75.111, M75.112, M75.120, M75.121, M75.122. From this index date, we constructed an episode of care that covered 365 days before and 90 days after a patient’s index date.

**Figure 1.**
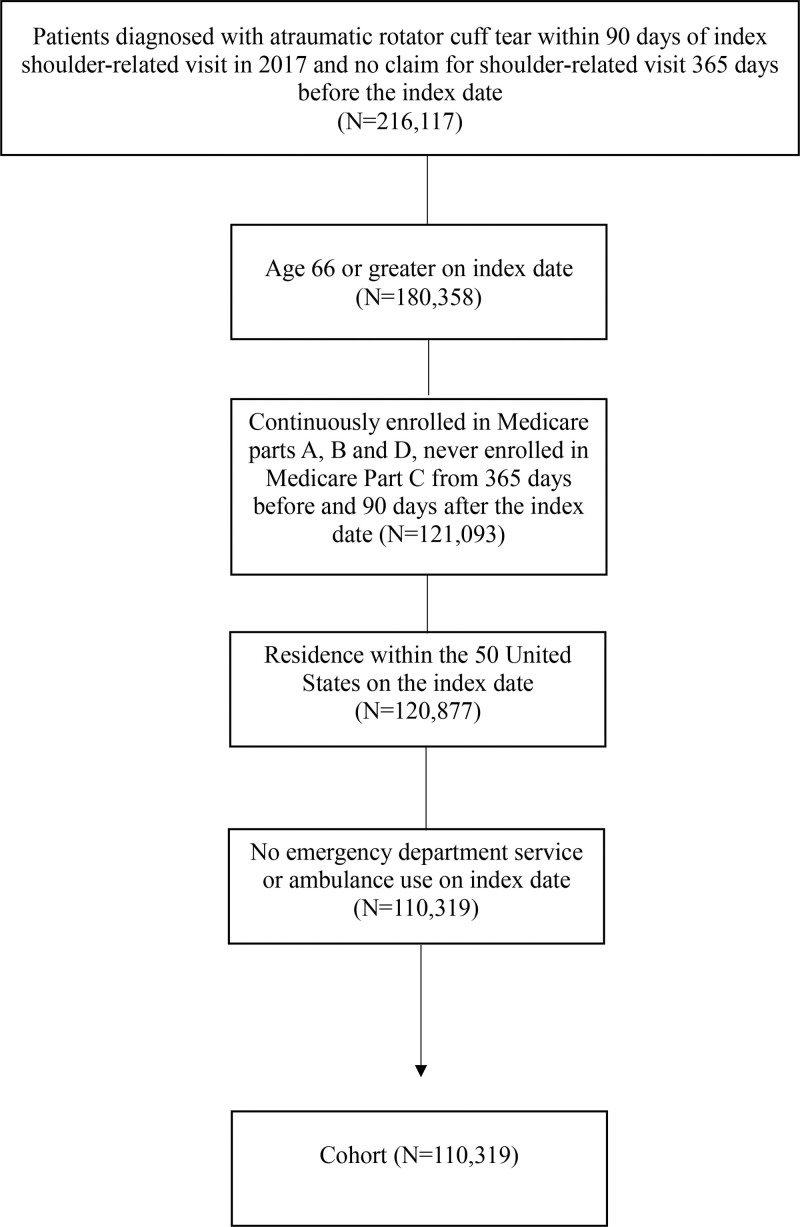
Cohort after applying inclusion criteria: Flow chart.

Additional inclusion criteria included continuous enrollment in Medicare parts A, B and D and no enrollment in Medicare part C from 365 days before through 90 days after the index date, minimum age of 66 years on the index date to ensure at least 12 months of prior claims data for accurate measurement of baseline covariates, given that individuals typically gain Medicare eligibility at age 65, and residence in the United States on the index date. To focus on nontraumatic cases, we excluded patients with emergency department services or an ambulance transfer on the index date. Figure 1 details the sample inclusion criteria and the number of patients following application of each criterion to construct our final analytical sample.

## 3. Variables

### 3.1. Dependent variables

We included 2 different dependent variables that encompassed distinct earlier PT definitions. First, we categorized a patient as receiving “PT first” if the patient saw only a physical therapist on the index date.^[24]^ If the patient saw any other provider in addition to a physical therapist on the index date, they were excluded from this definition. Second, we designated a patient as receiving “early PT” if the patient saw a physical therapist within 30 days of the index date, regardless of whether they sought care from another provider during this period. The PT-first cohort is therefore a subset of the early PT cohort. We adopted this 30-day cutoff for early PT use based on published literature.^[25]^

To identify whether a provider is a physical therapist, we obtained data from the Medicare Provider Practice and Specialty (MD-PPAS) 2017 file. The MD-PPAS file assigns Medicare providers to Centers for Medicare & Medicaid Services provider specialty classification.^[26]^ After merging the MD-PPAS file with the Medicare claims files, we identified providers with specialty code “65” as physical therapists. If the provider specialty was unavailable in the MD-PPAS file for a provider, we used the National Provider Identification file from 2017 to supplement our identification of physical therapists using the taxonomy code “225100000X.”

### 3.2. Independent variables

American Physical Therapy Association classifies state direct access laws into 3 categories: unrestricted, provisional, and limited.^[19]^ We cross-checked the APTA classification with published literature^[27]^ and selected state-level physical therapy associations to ensure data accuracy. In 2017, 18 states had unrestricted access, 26 states and District of Columbia had provisional access, and 6 states had limited access to PT (see Fig. B, Supplemental Digital Content).^[18]^ These different state direct access laws affected the ease with which patients could see a physical therapist earlier in the treatment process. Patients in states with *limited access* required a prior relevant medical diagnosis from a physician or other specified clinician and a physician referral prior to accessing PT. Patients in states with *provisional access* faced some restrictions, including limitations on the duration or number of visits without a prior physician referral for PT. For example, in New York, a provisional access state, a physical therapist can treat a patient for up to the earlier of 10 visits or 30 days, after which the patient must obtain a physician referral. Finally, states with *unrestricted access* allow patients to receive treatment from a physical therapist with no legal restrictions or limitations whatsoever. We verified that state direct access policy categories did not change during the study’s policy-assignment window (2016 and 2017), so beneficiaries were linked to a stable policy designation during baseline and index-year measurement.

### 3.3. Control variables

We included as covariates patient demographic characteristics available in Medicare’s 2017 Master Beneficiary Summary file. Patient-level variables included age on the index date, sex, race, and Medicaid dual-eligibility status during the month of the index visit. Patient health status was measured using the Charlson Comorbidity Index (CCI)^[28-30]^ and the Frailty Risk Index (FRI)^[31]^ using information from 365 days prior to the index date. CCI is a weighted index based on ICD-10 diagnosis codes that reflects the number and severity of comorbid conditions. A score of 0 indicates no comorbidities. The FRI measures frailty among older adults using factors such as use of a cane, walker, or wheelchair, with a score of 0 indicating non-frailty.

To capture differences in patient healthcare-seeking behaviors, we also calculated patients’ total normalized Medicare claims in the 365-day period prior to the index date and placed them into quartiles as additional measures.^[32]^ We recorded whether patients had a “prior use of PT” for non-shoulder-related condition in the 365-day period before the index date to control for patient preference for the use of PT and patient PT-seeking behavior. Lastly, to account for variation in workforce availability, we controlled for the state-level supply of physical therapists per 1,00,000 residents. Population estimates were obtained from the 2017 American Community Survey and the number of physical therapists in each state was derived from the 2017 Area Health Resource File.^[33]^

### 3.4. Analytical approach

We first summarized patient characteristics by use of PT and by types of state direct access laws to assess whether systematic differences existed based on these measures. Differences were assessed using 2-sample *t* tests or ANOVA for continuous variables, as appropriate, and chi-square tests for categorical variables. We then ran separate multivariable logistic regressions to assess the association between the earlier PT use variables and state direct access laws, controlling for the covariates identified above. In our multivariable analyses for aim 1, limited-access states alone served as the reference category; we estimated contrasts for provisional versus limited and unrestricted versus limited. We also conducted a secondary analysis restricted to states with provisional or unrestricted access to address current policy relevance. Specifically, because limited-access laws are no longer in effect nationwide (i.e., all states now fall into either provisional or unrestricted access), we estimated the unrestricted versus provisional contrast to reflect the primary policy distinction in the contemporary direct-access landscape. Finally, we also ran an analysis excluding those individuals who were dually enrolled in Medicaid and Medicare to remove potential confounding from coverage of PT under Medicaid plans. Results are presented as odds ratio (OR) with accompanying 95% confidence intervals (95% CI). We used SAS software 9.4 (Cary) to conduct the analyses. A *P* value of <.05 was deemed statistically significant.

### 3.5. Ethics approval

This study was exempt from the University of South Carolina Institutional Review Board review under Category 4 of 45 CFR 46.101(2)(b) because it was based on existing deidentified secondary data. Information in the data was recorded in such a manner that subjects cannot be identified, directly or through identifiers linked to the subjects.

## 4. Results

In Table 1, we present the summary statistics of key variables in our analytic sample. We had a total of 1,10,319 individual patients after applying our inclusion and exclusion criteria. Overall, 1.7% of patients with ARCT used PT first and 14.3% used early PT. In our data, 46.1% of the patients were male, 89.2% were White, and 7.2% were dually eligible for Medicare and Medicaid. Patients were most commonly aged 70 to 75 years (36.3%), followed by 66 to 69 years (27.9%). Thirty percent (30%) of the patients had a CCI score of 0 (30.0%) and 64.5% had an FRI score of 0. Finally, 14% of the patients had seen a physical therapist in the 365-day period before the index date for a non-shoulder condition capturing patient PT-seeking behavior.

**Table 1:** Baseline descriptive characteristics of study sample of patients with atraumatic rotator cuff tear by use of physical therapy.

	Overall	Panel A: PT first			Panel B: Early PT		
		No	Yes	*P*-value	No	Yes	*P*-value
Number of patients	1,10,319	1,08,433 (98.3%)	1886 (1.7%)		94,594 (85.3%)	15,770 (14.3%)	
Direct access laws							
Limited	16.0	16.2	8.5	**<.001**	16.4	13.6	**<.001**
Provisional	62.7	62.6	67.9	**<.001**	62.2	65.1	**<.001**
Unrestricted	21.3	21.3	23.5	**.02**	21.3	21.3	.88
Race/ethnicity							
Asian	1.4	1.4	2.4	**<.001**	1.4	1.5	.11
Black	4.5	4.5	3.2	**<.001**	4.7	2.8	**<.001**
Hispanic	1.3	1.3	1	.24	1.3	0.7	**<.001**
Other	3.6	3.6	5.4	**<.001**	3.5	4.4	**<.001**
White	89.2	89.3	88.0	.08	89.0	90.5	**<.001**
Male	46.1	46.2	40.1	**<.001**	46.2	45.9	.47
Age group (yr)							
66–69	27.9	27.9	27	.39	27.8	28.4	.10
70–75	36.3	36.3	35.4	.40	36.0	37.9	**<.001**
76–79	15.8	15.8	16.4	.47	15.8	15.9	.83
80–85	13.1	13.1	14.8	**.03**	13.2	12.9	.27
86+	6.8	6.8	6.4	.40	7.2	4.9	**<.0001**
Frailty Risk Index							
0	64.5	64.4	68.2	**<.001**	63.5	70.5	**<.001**
1	22.9	22.9	22.4	.72	23.1	21.4	**<.001**
2	7.4	7.4	6.2	**.06**	7.7	5.5	**<.001**
3	5.2	5.3	3.0	**<.001**	5.7	2.6	**<.001**
Charlson Comorbidity Index							
0	30.0	30.0	32.8	**<.001**	29.3	34.7	**<.001**
1	21.2	21.2	21.6	.69	21.1	22.0	**.01**
2	15.6	15.6	16.8	.15	15.6	16.0	.20
3	11.5	11.5	9.9	**.03**	11.6	10.5	**<.001**
4+	21.7	21.7	18.9	**<.001**	22.5	16.8	**<.001**
Dual eligible for Medicare and Medicaid	7.2	7.2	6.8	.50	7.8	4.1	**<.001**
Mean number of physical therapists per 1,00,000 population	85.8	85.7	90.3	**<.001**	85.4	88.4	**<.001**
Prior use of PT	14.0	13.6	34.0	**<.001**	12.6	22.4	**<.001**
Pre-365 costs*	$12,400	$12,421	$11,083	**<.001**	$12,774	$10,147	**<.001**

Statistically significant results (*P* < .05) in bold.

PT = physical therapy.

* Total Medicare reimbursements by patient over the 365-day period prior to index date.

Panels A and B of Table 1 show that patients with ARCT using PT first or early PT tend to differ on observable characteristics, justifying a need to control for these differences in our analyses. On average, compared to non-PT first patients, PT first patients were more likely to be female, Asian, have an FRI score or CCI score of 0. Early PT patients were more likely to be white or aged 66 to 69 years. Table 2 presents unadjusted differences in PT use across states with different laws. In the unadjusted descriptive statistics, we see that states with limited access to PT had lower rates of PT first or early PT use. However, the table suggests that underlying differences in populations across states with different legal environments require multivariable regressions to account for these variations.

**Table 2:** Baseline descriptive characteristics of study sample of patients with atraumatic rotator cuff tear by direct access laws, 2017.

	Overall	Direct access laws			*P*-value
		Limited	Provisional	Unrestricted	
Number of patients	1,10,319	17,690	69,117	23,512	
PT first	1.7	0.9	1.9	1.9	**<.001**
Early PT	14.3	12.2	14.9	14.3	**<.001**
Race/ethnicity					**<.001**
Asian	1.4	0.9	1.7	0.9	
Black	4.5	5.5	4.5	3.7	
Hispanic	1.3	1.7	1.3	0.8	
Other	3.6	2.3	3.8	4.1	
White	89.2	89.6	88.7	90.4	
Male	46.1	44.9	45.8	48.0	**<.001**
Age group (yr)					**<.001**
66–69	27.9	27.2	27.6	29.4	
70–75	36.3	36.5	36.1	36.6	
76–79	15.8	16.2	15.8	15.5	
80–85	13.1	13.3	13.4	12.1	
86+	6.8	6.8	7.0	6.4	
Frailty Risk Index					**<.001**
0	64.5	63.9	64.7	64.5	
1	22.9	22.5	22.8	23.4	
2	7.4	7.6	7.4	7.3	
3	5.2	6.0	5.1	4.9	
Charlson Comorbidity Index					**<.001**
0	30	29.0	29.2	33.2	
1	21.2	21.4	21.2	21.2	
2	15.6	15.6	15.7	15.4	
3	11.4	11.1	11.7	10.9	
4+	21.7	22.9	22.2	19.3	
Dual eligible for Medicare and Medicaid	7.2	6.4	7.9	6	**<.001**
Prior use of PT	14.1	10.4	14.6	15.1	**<.001**
Mean number of physical therapists per 1,00,000 population	85.8	77.2	84.3	96.8	**<.001**
Pre-365 costs*	$12,400	$13,083	$12,645	$11,158	**<.001**

Statistically significant results (*P* < .05) in bold.

PT = physical therapy.

* Total Medicare reimbursements by patient over the 365-day period prior to index date.

### 4.1. Regression results

Table 3, panel A shows results from a multivariable logistic regression assessing the association between using PT first and state direct access laws, controlling for patient sociodemographic and clinical characteristics. The adjusted odds of using PT first were significantly higher for patients residing in unrestricted states (OR = 1.8, 95% CI: 1.5–2.1, *P*-value < 0.001) and for patients residing in provisional access states (OR = 1.6, 95% CI: 1.3–2.1, *P*-value < 0.001), compared to patients in states with limited access. The results for early PT use as the dependent variable are presented in Table 3, panel B. These estimates reveal that patients residing in states with provisional access have higher adjusted odds of early PT (OR = 1.1, 95% CI: 1.1–1.3, *P*-value < .001) compared to those in states with limited access. However, no significant difference was observed in early PT use between states with unrestricted versus limited access (Table 3, panel B).

**Table 3:** Logistic regression results showing the association between state direct access laws and use of physical therapy among Medicare patients with atraumatic rotator cuff tears.

	Panel A: PT first	Panel B: Early PT
	OR [95% CI]	OR [95% CI]
Direct access laws		
Limited	Reference	
Provisional	**1.8 [1.5–2.1]**	**1.2 [1.1–1.3]**
Unrestricted	**1.6 [1.3–2.0]**	0.9 [0.9–1.04]
Sex		
Female	Reference	
Male	**0.8 [0.7–0.9]**	1.0 [0.9–1.0]
Race/ethnicity		
White	Reference	
Asian	**1.6 [1.2–2.2]**	**1.3 [1.1–1.5]**
Black	0.8 [0.6–1.1]	**0.7 [0.6–0.7]**
Hispanic	0.9 [0.6–1.6]	**0.8 [0.7–1.1]**
Other	**1.5 [1.2–1.9]**	**1.3 [1.1–1.4]**
Age group (yr)		
66–69	Reference	
70–75	1.0 [0.9–1.1]	1.0 [0.9–1.1]
76–79	1.1 [0.9–1.3]	1.0 [0.9–1.1]
80–85	**1.2 [1.0–1.4]**	1.0 [0.9–1.1]
86+	1.1 [0.9–1.3]	**0.7 [0.6–0.8]**
Charlson Comorbidity Index		
0	Reference	
1	0.9 [0.8–1.0]	**0.9 [0.8–1.0]**
2	0.9 [0.8–1.2]	**0.9 [0.8–1.0]**
3	**0.8 [0.7–1.0]**	**0.9 [0.7–1.0]**
4 or more	0.9 [0.8–1.0]	**0.8 [0.7–0.9]**
Frailty Risk Index		
0	Reference	
1	**0.9 [0.7–0.9]**	**0.82 [0.8–0.9]**
2	**0.8 [0.6–0.9]**	**0.7 [0.6–0.8]**
3 or more	**0.6 [0.5–0.8]**	**0.5 [0.4–0.6]**
Dual eligible for Medicare and Medicaid*	0.9 [0.8–1.2]	**0.6 [0.5–0.7]**
Pre-365 cost quartiles†		
Cost pre (Q1)	Reference	
Cost pre (Q2)	1.0 [1.0–1.2]	1.0 [0.9–1.1]
Cost pre (Q3)	1.2 [1.0–1.3]	1.0 [0.9–1.1]
Cost pre (Q4)	0.9 [0.7–1.0]	**0.8 [0.8–0.9]**
Supply of physical therapists per 1,00,000 population (quartiles)		
Q1	Reference	
Q2	0.9 [0.7–1.0]	0.9 [0.9–1.0]
Q3	1.1 [0.9–1.2]	1.0 [0.9–1.1]
Q4	**1.5 [1.3–1.7]**	**1.4 [1.3–1.5]**
Prior use of PT*	**3.2 [2.9–3.5]**	**2.1 [1.9–2.2]**

Model was adjusted for all covariates.

Statistically significant results (*P* < .05) in bold.

CI = confidence interval, OR = odds ratio, PT = physical therapy.

* Reference group: not dually eligible for Medicaid and no use of PT 365 days prior to the index date.

† Total Medicare reimbursements by patient over the 365-day period prior to index date captured into 4 equal quartiles.

While not the primary focus of this study, our covariates also show several patterns suggesting differential preference for PT use. We found, for example, that Asians (OR = 1.6, 95% CI: 1.2–2.2), patients identified as other race, (OR = 1.5, 95% CI 1.2–1.9), patients aged 80 to 85 years (OR = 1.2, 95% CI: 1.0–1.4), and patients who had prior use of PT (OR = 3.2, 95% CI: 2.9–3.5) were significantly more likely to use PT first. On the other hand, frailer and sicker patients, i.e., patients with higher FRI scores (i.e., FRI 1; OR = 0.9, 95% CI: 0.7–0.9) or CCI of 3 (OR = 0.8, 95% CI: 0.7–1.0) had significantly lower odds of using PT first.

When we restricted the analysis only to states with provisional or unrestricted direct access laws (i.e., excluding states that had limited access), we did not observe any significant differences in earlier PT use (both PT first and early PT). Second, when we excluded individuals dually eligible for Medicare and Medicaid, our results remained similar to what we observed in our overall cohort (Table 4).

**Table 4:** Logistic regression results showing the association between unrestricted and provisional access states and for only Medicare patients (excluding dually enrolled patients).

	Physical therapy first	Early physical therapy
	OR [95% CI]	OR [95% CI]
Model 1: Excluding individuals in states with limited access in 2017		
Provisional	Reference	Reference
Unrestricted	0.9 [0.8–1.0]	0.9 [0.8–1.1]
Model 2: Excluding individuals dually enrolled for Medicare and Medicaid		
Limited	Reference	Reference
Provisional	**1.8 [1.5–2.1]**	**1.1 [1.1–1.3]**
Unrestricted	**1.7 [1.4–2.1]**	1.0 [1.0–1.1]

Model controls for sex, age categories, race categories, Frailty Index, Charlson Comorbidity Index, use of physical therapy in the baseline period, baseline expenditure, and physical therapist per capita.

Statistically significant results (*P* < .05) in bold.

CI = confidence interval, OR = odds ratio.

## 5. Discussion

Our findings show that provisional and unrestricted state direct access laws are associated with greater odds of earlier use of PT among patients diagnosed with ARCT. Specifically, patients in states with unrestricted or provisional access had approximately 1.8- and 1.6-fold higher odds, respectively, of using PT first compared with those in limited-access states. Additionally, compared with patients in states with limited access, patients in states with provisional access had 1.2 times the odds of using PT first. We also show that patients in states with unrestricted access did not have higher odds of earlier PT as compared to those in states with provisional access.

State direct access laws shape PT utilization patterns, however they are not universally accepted. Proponents of these laws argue that more direct access can increase the use of PT, reduce healthcare utilization, and improve patient satisfaction by enabling earlier intervention.^[24,34-36]^ On the other hand, opponents raise concerns about the potential overutilization of resources and underdiagnosis, especially for complex cases that may require physician oversight.^[36]^ Our findings can begin to help resolve this debate.

Our study is the first, to our knowledge, to isolate the associations between state direct access laws and earlier use of PT. The findings that provisional and unrestricted direct access is associated with earlier PT use reinforce the steps states have already taken to expand direct access to PT. However, the finding that unrestricted access was not associated with greater odds of earlier PT use compared to provisional access suggests, at least preliminarily, that no further gains may be obtained from providing unrestricted access to PT. Pending additional studies confirming these results using more recent data, our study results suggest that provisional access may provide a balanced approach to address some of the concerns of both proponents and opponents of liberal access to physical therapists. Additionally, APTA’s 2025 report shows that direct access to PT continues to be a priority across the United States.^[37]^ Although most states now allow at least provisional direct access, APTA notes that regulations, insurance policies, and patient awareness still affect care. Our findings overall highlight that differences in state-level policies continue to affect utilization.

In addition, factors other than state direct access laws also contribute to PT use. We show, for example, that Black and Hispanic patients and those dually eligible for Medicare and Medicaid are less likely to use PT, suggesting that structural, cultural, financial, or medical barriers remain even after legal barriers are removed. For example, patients dually eligible for Medicare and Medicaid may be more likely to be in poorer health,^[38-41]^ and therefore may not be able to receive PT. In addition, some patients may face greater financial constraints paying for out-of-pocket medical expenses,^[40,42]^ thereby limiting access to PT. Finally, we found that older and frailer and sicker patients, as indicated by high FRI or CCI scores, are less likely to use PT first or early PT.

## 6. Limitations

The study has a few limitations. First, while we provided an empirical setting in which we design-control for patients and benefit-plan, our results may not generalize to commercially insured or younger populations, which warrant a separate study. Second, as with all claims-based analyses, we could not directly measure certain clinical factors that may influence PT timing, including symptom severity (e.g., pain intensity or functional limitation) and imaging findings (e.g., tear size). If unmeasured severity differs systematically across policy environments, residual confounding is possible. To mitigate this concern, we adjusted for multiple proxies of health status and care-seeking (including comorbidity, frailty, and prior utilization). Moreover, we would not expect clinical severity or tear size to vary systematically by state direct access policy category, which may limit the potential for this unmeasured confounding to drive the observed associations. Third, our study only assesses the association between state direct access laws and PT utilization patterns. Subsequent studies are needed to examine the potential impact of these laws on health outcomes. Finally, we were not able to account for geographic differences across the states. However, we did attempt to account for variation in workforce availability by controlling for the state-level supply of physical therapists per 1,00,000 residents.

In conclusion, we show that state laws permitting provisional or unrestricted access are associated with higher odds of earlier PT use compared to states with limited access among Medicare FFS patients with ARCT. Using data from 2017, we also found that unrestricted states did not have greater use of earlier PT than provisional states. These findings suggest that past laws may have helped encourage earlier use of PT among Medicare FFS patients. However, given that all states today have either provisional or unrestricted direct access, our additional analyses showing no difference between these 2 types of states require updated substantiation to assess the benefits of further liberalizing state direct access laws. Future research should also investigate whether these differences in care-seeking behavior may also improve health outcomes or reduce healthcare expenditures. Establishing the causal effect of policy would require a within-state pre–post design around policy liberalization or quasi-experimental methods such as difference-in-differences or instrumental variables, which we recommend for future research. Further, now that all states have either provisional or unrestricted access to PT, future studies could evaluate changes in PT use (both PT first and early PT) following the transition from limited to provisional access to better understand the impact of the direct access laws.

## Acknowledgments

We acknowledge and thank the Center for Effectiveness Research in Orthopedics (CERorth) for providing the Medicare data files to complete our research study.

## Author contributions

**Conceptualization:** Dakshu Jindal, John M. Brooks, Brian K. Chen.

**Data curation:** Dakshu Jindal, John M. Brooks, Brian K. Chen.

**Formal analysis:** Dakshu Jindal, John M. Brooks, Brian K. Chen.

**Funding acquisition:** Dakshu Jindal, John M. Brooks, Brian K. Chen.

**Investigation:** Dakshu Jindal.

**Methodology:** Dakshu Jindal, John M. Brooks, Nicole L. Hair, Adam D. Lutz, Brian K. Chen.

**Project administration:** Dakshu Jindal.

**Resources:** Dakshu Jindal, John M. Brooks.

**Software:** Dakshu Jindal.

**Supervision:** Dakshu Jindal, John M. Brooks, Brian K. Chen.

**Validation:** Dakshu Jindal, Nicole L. Hair, Brian K. Chen.

**Visualization:** Dakshu Jindal.

**Writing – original draft:** Dakshu Jindal, John M. Brooks, Nicole L. Hair, Adam D. Lutz, Brian K. Chen.

**Writing – review & editing:** Dakshu Jindal, John M. Brooks, Nicole L. Hair, Adam D. Lutz, Brian K. Chen.




