# Supplementary figures and images for: The association between state direct access laws and earlier use of physical therapy among Medicare patients with rotator cuff tears: A retrospective cohort study
Source: Medicine (Baltimore). 2026 May 8;105(19):e48584. doi: 10.1097/MD.0000000000048584 (PMC13166533; doi:10.1097/MD.0000000000048584)

Supplementary Figure A: Cohort Identification


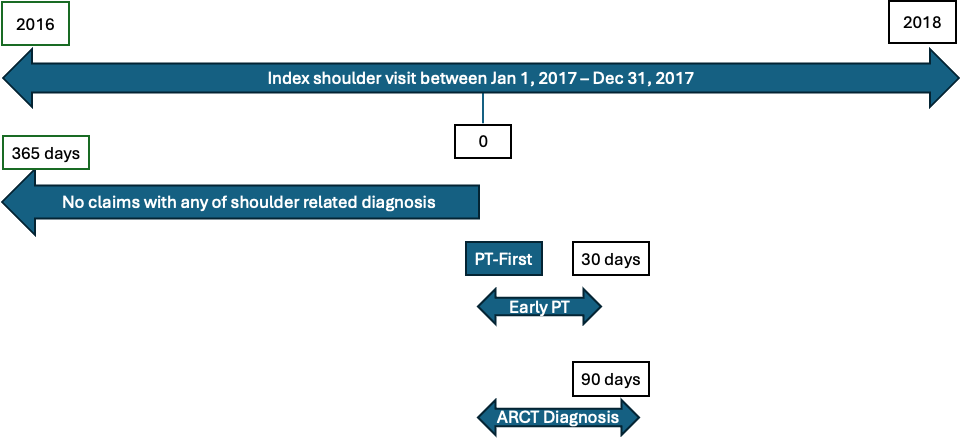

Supplement: Supplementary file 1 [file medi-105-e48584-s001.docx]
